# A community pharmacy-based program to enhance adherence to adjuvant endocrine therapy among breast cancer survivors (PACHA): protocol for a pilot cluster-randomized controlled trial

**DOI:** 10.1186/s40814-025-01676-8

**Published:** 2025-07-14

**Authors:** Julie Lapointe, Laurence Guillaumie, Anne Dionne, Lyne Lalonde, Julie Lemieux, Michel Dorval, Hermann Nabi, Martine Lemay, Line Guénette, Jason Robert Guertin, Benoît Mâsse, Sophie Lauzier

**Affiliations:** 1 CHU de Québec-Université Laval Research Center, Québec, QC Canada; 2 Faculty of Nursing, Université Laval, Québec, QC Canada; 3 Faculty of Pharmacy, Université Laval, Québec, QC Canada; 4 Centre Des Maladies du Sein, CHU de Québec-Université Laval, Québec, QC Canada; 5 Faculty of Pharmacy, University of Montreal, Montreal, QC Canada; 6 CISSS Chaudière‐Appalaches Research Center, Lévis, Canada; 7 Department of Social and Preventive Medicine, Faculty of Medicine, Université Laval, Québec, Canada; 8 CHU Sainte-Justine Research Center, University of Montreal, Montreal, QC Canada

**Keywords:** Adjuvant endocrine therapy, Breast cancer, Community pharmacy services, Medication adherence, Motivational interviewing, Professional education, Pilot study

## Abstract

**Background:**

Adjuvant endocrine therapy (AET) is an oral treatment prescribed for 5 to 10 years to women with hormone-sensitive breast cancer. Despite the benefits of AET for reducing breast cancer recurrence, suboptimal adherence is common. Community pharmacists can play a role in supporting women with this treatment, given their frequent encounters with patients, access to refill information, and expertise in managing side effects. The goal of this pilot study is to assess the acceptability and feasibility of implementation, and preliminary effects of the PACHA program, a community pharmacy-based program designed to support women who are prescribed AET. Another goal is to assess the feasibility of a large-scale randomized controlled trial (RCT).

**Methods:**

This is a pilot cluster-RCT using mixed-methods. A cluster consists of a pharmacy, its pharmacists, and its patients with an AET prescription in the last 30 months. Pharmacies will be recruited through targeted advertisement. Participating pharmacies will be randomized 1:1 to two groups (*n* = 33 pharmacies per group). In the first group (control), pharmacists will provide usual services to women. In the second group (intervention), pharmacists will complete web-based training and perform consultations using a standardized guide based on motivational interviewing principles and evidence-based online strategy sheets to cope with AET side effects. Women in the intervention group will have access to a website featuring video modules on AET, strategies for managing side effects, testimonials from women who have had an AET, and a list of resources. Acceptability and feasibility indicators, as well as psychosocial factors expected to influence AET adherence and treatment experience, will be collected at baseline, during, and at the end of the 6-month follow-up using online questionnaires, study data logs, and pharmacy claim records. Semi-structured interviews will be conducted to explore participants’ experiences with the program.

**Discussion:**

Results will help to refine the program and, if the results support this, to design a full-scale cluster-RCT to assess the program’s effect on 5-year adherence and costs. If effective, this program could fill a gap in breast cancer supportive care and contribute to reducing cancer burden by improving survivorship experience and survival.

**Trial registration:**

This trial has been approved by the Research Ethics Board of the CHU de Québec-Université Laval (MP-20–2023-6625) and registered at Clinicaltrials.gov (NCT05887102) on 2023–05-24, https://classic.clinicaltrials.gov/ct2/show/NCT05887102, prior to beginning the study. Protocol version 1 is dated as 2022–12-12.

## Background

Breast cancer is the most common cancer in women worldwide [1]. Adjuvant endocrine therapy (AET) (i.e., tamoxifen or aromatase inhibitors) is an oral medication prescribed to women with hormone-sensitive breast cancer (60% to 75% of cases [2, 3]) to reduce recurrence and mortality risks [4]. AET must be taken daily for 5 to 10 years after other treatments, such as surgery, radiotherapy, and chemotherapy [5, 6]. Five years of tamoxifen reduces recurrence rates during the first 5 years by 47% and breast cancer mortality by 30% 15 years after diagnosis [4]. The efficacy of aromatase inhibitors is even greater in post-menopausal women [7].

AET can be challenging for patients who have to self-manage their treatment [8, 9]. Despite the widely recognized clinical benefits of AET, suboptimal AET adherence (i.e., non-persistence or sub-optimal implementation) is common [10–13]. A meta-analysis estimated that 47% and 31% of women prescribed tamoxifen and aromatase inhibitors, respectively, did not persist with their AET for 5 years [13]. Another systematic review indicated that 28% to 59% of patients collected less than 80% of their prescribed doses [10]. Non-adherence has been found to be associated with an increased risk of breast cancer recurrence and mortality [14, 15] and higher costs for the healthcare system [16]. Several factors must be considered to improve AET adherence, such as the management of AET side effects (e.g., hot flashes, musculoskeletal pain, and insomnia), the provision of sufficient information on AET and the availability of supportive patient-provider relationships and communication [17–21].

The development and evaluation of innovative approaches for enhancing AET adherence are needed to attain the full benefits of this treatment. A recent meta-analysis reported that existing AET adherence interventions provided positive but small effects [22]. However, none of these interventions tested the potential impact of community pharmacists on AET adherence. Nevertheless, community pharmacists are pharmacotherapy experts and can play a key role in enhancing AET adherence. They frequently interact with patients, have access to medication data to detect non-adherence, and have the expertise to suggest and implement strategies to optimize medication use and overcome side effects. This is especially true in several jurisdictions where the role of pharmacists has evolved over the past few decades from drug distribution to performing more clinical interventions [23].

To optimize AET adherence, we co-designed the PACHA program *(programme en Pharmacie pour l’ACcompagnement des femmes ayant de l’Hormonothérapie Adjuvante—*in English, *Pharmacy program for supporting women undergoing adjuvant endocrine therapy)*. PACHA is, to our knowledge, the first community pharmacy-based intervention specifically aimed at enhancing AET adherence among women with non-metastatic breast cancer [24]. As a first step, we designed a pilot study to determine if involving community pharmacists to support breast cancer survivors with AET is acceptable, feasible, and promising. More specifically, this pilot study evaluates the program’s (1) acceptability from the pharmacist’s and patient’s perspective, (2) feasibility of implementation, (3) preliminary effects on factors expected to influence AET adherence according to our conceptual model (intention to adhere to AET, AET knowledge, attitude, perceived social support, perceived behavioral control, anticipated regret, coping planning, and fear of recurrence), and (4) feasibility and required sample size for a large-scale randomized controlled trial (RCT).

## Methods

### Study design and setting

This is a pilot cluster-RCT with a 6-month follow-up, using mixed methods (quantitative and qualitative) and recruiting in the province of Québec, Canada. Following the Medical Research Council framework for complex interventions [25], the PACHA program and study procedures will be pilot-tested to guide decisions regarding the development of a large-scale, cluster-RCT assessing the effects of the program on 5-year AET adherence and healthcare system costs. The need for a pilot study lies in the novelty of the program and the anticipated challenges related to implementing interventions and conducting a RCT in community pharmacies [26]. A cluster is defined as a community pharmacy, its pharmacists, and its recruited patients. Cluster randomization is necessary to prevent between-group contamination (i.e., pharmacists in the control group exposed to PACHA training and tools). The PACHA program will be compared to usual community pharmacy practice. Each cluster will be followed for 6 months after their inclusion in the study (randomization). See Table 1 for the study timetable. This trial has been approved by the Research Ethics Board of the CHU de Québec-Université Laval (MP-20–2023-6625) and registered at Clinicaltrials.gov (NCT05887102) on 2023–05–24, prior to beginning the study. The study is presented according to the SPIRIT recommendations [27].

**Table 1:**
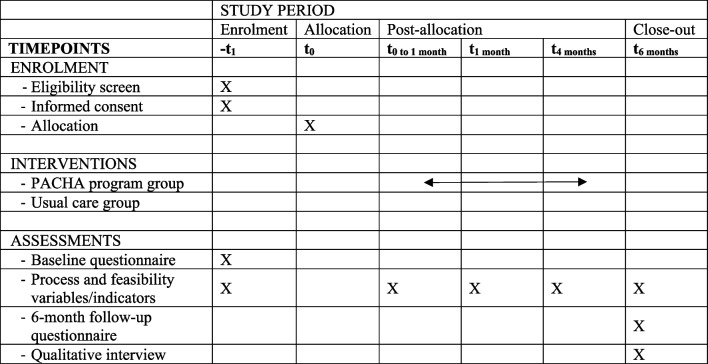
Study timetable according to SPIRIT guidelines for presenting the study’s phases

### Intervention

**Fig. 1:**
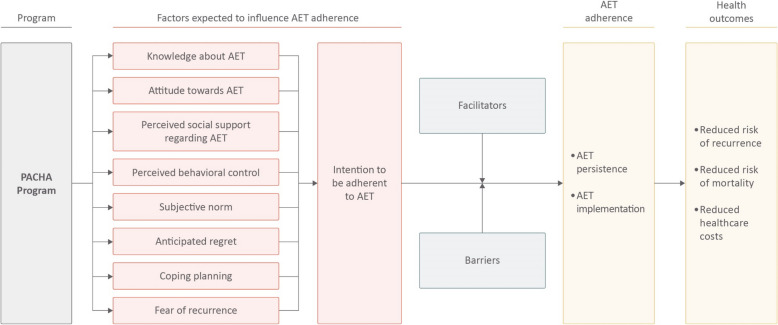
Conceptual model guiding the intervention development and evaluation adapted from the Behavioral Model for Medication Adherence

Given the complexity of medication-taking behaviors, we opted for an adaptation of the Behavioral Model for Medication Adherence [28, 29] to guide the development and evaluation of the PACHA program [24] (see Fig. 1). This model is based on the Theory of Planned Behavior, one of the most cited and influential theoretical models for predicting behaviors [30, 31]. It was adapted for AET adherence and empirically validated in a qualitative study we conducted among 43 patients [32]. This model depicts the factors expected to influence AET adherence. In this model, AET adherence is influenced by the patient’s *behavioral intention*, i.e., the degree to which the patient intends to take the AET as prescribed. Numerous *barriers* (e.g., side effects) can hinder the translation of this intention into AET adherence. Conversely, *facilitators* (e.g., establishment of a routine) can help translate intention into AET adherence. *Intention* is, in turn, influenced by *AET knowledge, attitude*, *perceived social support*, and *perceived behavioral control*. *Attitude* refers to balancing the perceived advantages and disadvantages of adhering to AET. *Perceived social support* refers to the extent to which patients perceive that significant others and healthcare professionals support them in adhering to AET. *Perceived behavioral control* refers to whether the patients feel able to face barriers. In our previous research [32], anticipated regret (i.e., the feeling one would experience if a behavior was not adopted [33]), coping planning (i.e., strategies one would adopt to overcome potential barriers to action [34]), and fear of recurrence were also found to be important factors influencing AET adherence and were added to the model. In addition to intention, perceived behavioral control is also hypothesized to directly influence AET adherence.

The PACHA program was co-designed by a planning group (*n* = 11) of researchers (expertise in medication adherence, health interventions, breast cancer), a hospital pharmacist specializing in oncology, patients who were prescribed an AET, and community pharmacists. We followed the six steps of Intervention Mapping, an approach guiding the development, implementation, and evaluation of complex behavioral interventions in health promotion [35]. In *Step 1*, a conceptual model was drawn up based on a literature review on AET adherence [10, 12, 17, 36–39] and a qualitative study conducted by our team [32]. In *Step 2*, program objectives were formulated based on this conceptual model. The behavioral objective of the intervention is that “Each patient with a new prescription has an optimal use of AET”. Six different performance objectives were deemed essential to achieve this behavioral objective. Change objectives were defined at the intersection of factors influencing AET adherence arising from the conceptual model and performance objectives. In *Step 3*, the theoretical methods and practical applications to fulfil these objectives were selected. Principles of motivational interviewing (MI), a collaborative conversation style to reinforce a person’s motivation and commitment to change [40], were chosen and adapted for the pharmacist’s intervention. MI has a positive effect on drug adherence for chronic diseases [41, 42]. In community pharmacy practice, the potential of MI lies in its ability to be effectively implemented within the typically brief consultation time frames [43] and the fact that it is increasingly being taught in pharmacy faculties [44, 45]. In *Step 4*, the program sequence and tools were developed. In *Step 5*, an implementation plan was developed by creating a second matrix of change objectives. In *Step 6*, the evaluation plan and the pilot study were designed. The development of the program was guided by the idea that it should be low-cost, aligned with provincial pharmacy practice standards, brief and easy to integrate into pharmacy operations, and tailored to meet patients’ specific needs. Results of this Intervention Mapping process were published [24].

PACHA includes components for both the pharmacists and the patients. For the pharmacists, components are as follows: (1) web-based training eligible for continuing education credits about AET and AET consultations based on MI; and (2) standardized tools to guide their AET consultations (consultation guide based on MI principles and a series of evidence-based sheets of non-pharmacological and pharmacological strategies for coping with AET side effects). For the patients, it includes (1) web-based video modules about AET; (2) self-management strategies for coping with specific AET side effects; (3) testimonials from patients using an AET; and (4) a detailed list of resources for breast cancer survivors (e.g., national and provincial breast cancer societies or foundation website links and toll-free helplines). Table 2 provides a detailed description of the intervention (i.e., PACHA program) compared to usual care (i.e., usual care (UC)).

**Table 2:** Description of the PACHA program group and the usual care group

**PACHA program group**	**Usual care group**
**For the pharmacists : ** **An 85-minute web-based training **comprising three main sections: 1) a review of AET pharmacotherapy; 2) information on AET consultation based on the principles of motivational interviewing with video simulations; and 3) access to materials geared to participating patients (video modules about AET, self-management strategies for coping with specific AET side effects and list of resources). The training can be completed in one or several sessions in the weeks after randomization. Pharmacists who complete the training can request continuing education credits from their regulatory licensing body, the *Ordre des pharmaciens du Québec *(OPQ). **Standardized consultation guide **based on motivational interviewing principles to guide AET telephone consultations.This standardized consultation guide outlines the key steps that the pharmacist must cover during the AET consultation and follows the usual sequence of consultation in pharmacy. This consultation guide is to be used in the first month and 4 months after randomization, or more frequently according to the needs. **Evidence-based web sheets **describing (non)pharmacological strategies to cope with side effects (e.g. hot flashes) and other AET-related concerns (e.g. sexuality). **For the patients : ** **Web-based video modules **to inform patients about AET.These video modules cover how to and why take tamoxifen or an aromatase inhibitor, the possible side effects and strategies to cope with these side effects and, finally, medication intake and daily activities (e.g. diet). **Evidence-based web sheets **describing (non)pharmacological strategies to cope with side effects (e.g. hot flashes) and other AET-related concerns (e.g. sexuality) in a version adapted for the patient. **Testimonies** of patients who had breast cancer and an AET. **List of resources** available to patients who needs help in coping with side effects and other AET-related concerns.	**For the pharmacists and patients: ** Pharmacists and patients in the usual care (UC) group will not have access to the program during the study and will continue to provide/receive their usual services (e.g. AET information, suggestions to deal with side effects). Pharmacists will be offered the web-based training at the end of the study and patients will be provided with access to the website.

#### PACHA program pre-testing

PACHA program components were pre-tested in two steps. *Step 1*: community pharmacists (*n* = 7) received online access to the intervention components. Appropriateness, completeness, acceptability, and feasibility were assessed through semi-structured interviews. Participants reported an overall high appreciation of the web-based training and judged that the intervention tools were well-structured, relevant, and presented in an accessible manner. The main areas of improvement mentioned were (1) offering further explanation about how MI principles could enhance pharmacists’ interventions; (2) showing the different tools and their use in greater detail in the video simulations; and (3) making the program delivery more flexible when the pharmacy is very busy (e.g., making an appointment with the patient or doing consultations by telephone; strategies that are already used in current pharmacy practice). *Step 2:* we conducted a study using simulated consultations with community pharmacists (*n* = 3) who completed the web-based training and patients on an AET (*n* = 7). Patients received the program at the Université Laval’s mock pharmacy, which is used to train pharmacy students. An individual semi-structured interview on the experience with providing/receiving the program was conducted with participating pharmacists and patients. The program components were refined based on feedback and the post-pandemic context of pharmacy practice. Indeed, the PACHA program consultations were initially conceived to be offered face-to-face [24]. However, due to changes in pharmacy practice following the COVID-19 pandemic, and after consulting with community pharmacists, some program components were modified to be delivered online or via telephone, instead of face-to-face.

### Eligibility criteria

*Community pharmacies* are eligible to participate if (1) at least one pharmacist agrees to take charge of the project in their pharmacy and provides consent; and (2) at least one patient has initiated AET in the last 30 months in the pharmacy and provides consent. *Patients* are eligible if they (1) are a woman; (2) ≥ 18 years; (3) were diagnosed with a first non-metastatic, hormone-sensitive breast cancer (endocrine therapy may also be prescribed to patients with metastatic disease, but in this case, the patient takes the drug to treat active cancer and so, adherence issues may differ); (4) received an AET prescription for the first time in the last 30 months; (5) are fluent in French (78% of the Quebec population) [46]; (6) have internet access; and (7) provide consent. Excluded are patients who live in a residential facility where AET is not self-managed.

### Recruitment

Pharmacies, pharmacists, and patients will be recruited in the province of Québec. Pharmacists will first be invited to participate in the study through advertisements such as those on the social media platforms of the provincial pharmacy college, the *Ordre des pharmaciens du Québec* (OPQ) and Facebook groups for pharmacists. The research team will also reach out directly via phone, email, or in person. Interested pharmacists will be provided with study details, and pharmacy eligibility will be verified. Eligibility will be determined by confirming that at least one patient has initiated an AET in the last 30 months as a client in the pharmacy (verified by the pharmacists). If that is the case, interested pharmacists will be asked to invite other pharmacists in their pharmacy to participate, identify a pharmacist responsible for overseeing the study in their pharmacy, and inform the pharmacy owner of their intent to participate. In each pharmacy, a member of the pharmacy team will contact potentially eligible patients to provide a brief overview of the study and request their permission to be contacted by a research team member for further details. A research team member will verify eligibility criteria with each patient. If the patient is eligible and agrees to participate, a link to the consent form and baseline questionnaire will be sent. A consent form and a baseline questionnaire will also be sent to pharmacists agreeing to participate.

Participants for the qualitative part of the study will be selected at the end of the follow-up to reflect on different characteristics likely to influence the experience with AET and/or with the program (for example, for the patient: age, type of AET, time since AET initiation, satisfaction with the program or AET services received in pharmacy; for the pharmacists: sex, years of experience, use of the program components, and/or AET services dispensed). This selection will be performed based on the information from the questionnaires.

### Allocation and blinding

**Fig. 2:**
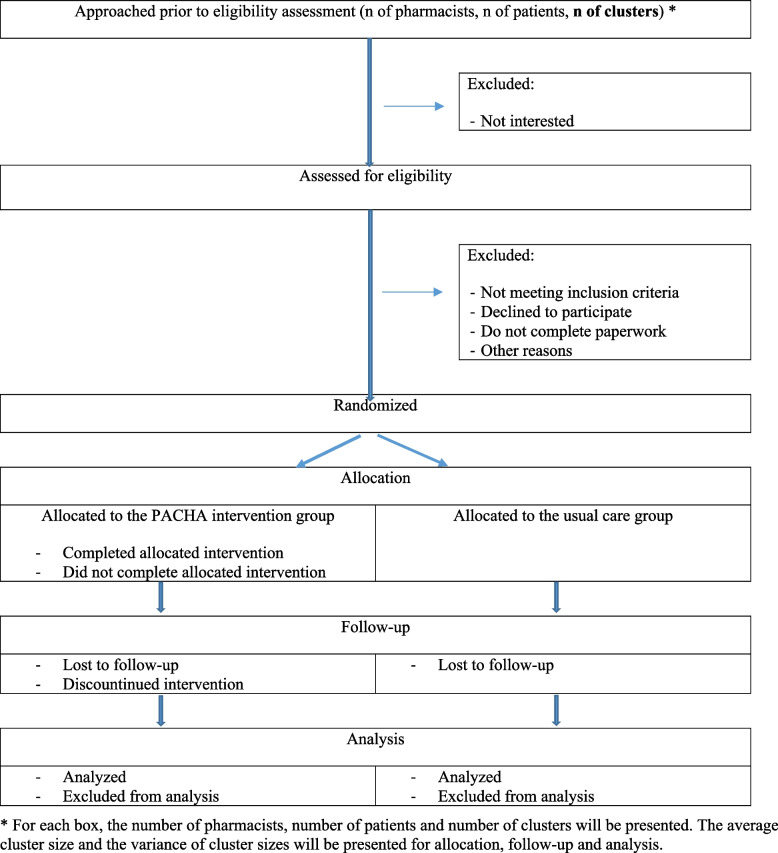
CONSORT diagram illustrating participants’ flow throughout this pilot cluster RCT

When all the consent forms and baseline questionnaires are completed, the pharmacy, its pharmacists and patients form a cluster and will be randomized (see Fig. 2). Each cluster will be randomly assigned to the PACHA program or the UC group using a 1:1 allocation ratio. A biostatistician, external to the study team, generated the randomization sequence using variable block sizes and prepared numbered, sealed envelopes to be opened in a strict order. After randomization, no other pharmacist or patient can be added to the cluster. Program components are available exclusively to pharmacies randomized to the PACHA program group. Pharmacists and patients are not blinded to the intervention, but data analyses will be conducted in a blinded fashion.

### Outcomes

The assessment plan for the study outcomes is presented in Table 3. *Program acceptability* will be assessed through participation and retention rates, uptake of program components, satisfaction, and participants’ perspectives on the relevance, acceptability, and perceived benefits of each program component. *Feasibility of the implementation of the PACHA program* will be evaluated using indicators based on the Framework for the Implementation of Services in Pharmacy [26], which includes indicators related to the implementation process, implementation impact, and implementation outcomes. The *implementation process* will be evaluated through the extent of program delivery (e.g., limited to some program components or its full capacity). The *implementation impact* will be assessed through factors and strategies influencing implementation at individual and environmental levels. *Implementation outcomes* will take into account reach and fidelity. Reach and fidelity indicators will include the timing of program components' uptake and delivery. Also, the dynamics of implementing the PACHA program in community pharmacies will be assessed with the noMAD questionnaire [47, 48] derived from the Normalization Process Theory of the implementation of complex health interventions [49].

**Table 3:** Assessment plan for the study outcomes

Outcome	Mode of measurement	Analysis of metric	Timeline			Group of participants assessed	
			Baseline	Throughout	End of study	Pharmacists	Patients
**Acceptability**							
Participation and retention rate	- Study data log	- Descriptive statistics		X	X	X	X
Uptake of program components	- Questionnaire - Website metrics	- Descriptive statistics		X	X	X	X
Satisfaction	- Questionnaire - Semi-structured interview	- Descriptive statistics - Thematic analysis			X	X	X
Perceived relevance, acceptability, and benefits	- Semi-structured interview	- Thematic analysis			X	X	X
**Feasibility of implementation**							
Extent of program delivery	- Questionnaire - Semi-structured interview - Website metrics	- Descriptive statistics - Thematic analysis		X	X	X	
Factors and strategies influencing implementation	- Semi-structured interview	- Thematic analysis			X	X	
Reach and fidelity: timing of program component uptake and delivery	- Questionnaire - Semi-structured interview - Website metrics	- Descriptive statistics - Thematic analysis		X	X	X	X
Dynamics of implementing complex interventions	- Questionnaire	- Descriptive statistics			X	X	
**Preliminary effects on factors expected to influence adjuvant endocrine therapy adherence**							
Intention to adhere to adjuvant endocrine therapy	- Questionnaire	- Mean change	X		X		X
Adjuvant endocrine therapy knowledge	- Questionnaire	- Mean change	X		X		X
Attitude towards adjuvant endocrine therapy	- Questionnaire	- Mean change	X		X		X
Perceived social support	- Questionnaire	- Mean change	X		X		X
Perceived behavioral control	- Questionnaire	- Mean change	X		X		X
Anticipated regret	- Questionnaire	- Mean change	X		X		X
Coping planning	- Questionnaire	- Mean change	X		X		X
Fear of recurrence	- Questionnaire	- Mean change	X		X		X
Side effects	- Questionnaire	- Mean change	X		X		X
Cognitive representations of the medication	- Questionnaire	- Mean change	X		X		X
Patient-provider relationship	- Questionnaire	- Mean score			X	X	X
Quality of life	- Questionnaire	- Mean change	X		X		
Adherence	- Questionnaire - Pharmacy claim records	- Descriptive statistics			X		X
**Feasibility of a large-scale cluster randomized controlled trial**							
Recruitment rates and channels	- Recruitment algorithm	- Descriptive statistics		X	X	X	X
Reasons for refusal	- Study data logs	- Descriptive statistics		X		X	X
Randomization process	- Recruitment algorithm	- Descriptive statistics		X		X	X
Cluster size	- Study data logs	- Descriptive statistics		X			
Frequency of contact between participants and research team	- Study data logs	- Descriptive statistics		X		X	X

Outcomes related to the *preliminary effects on factors expected to influence AET adherence*, such as intention to adhere to AET, AET knowledge, attitude, perceived social support, behavioral control, anticipated regret, and coping planning, will be assessed by questionnaire. We developed this questionnaire following the recommendations of the developers of the Theory of Planned Behavior [50] and our previous qualitative study [32]. The questionnaire was validated in a test–retest study among 67 patients with an AET, and each scale presented very good psychometric properties (Cronbach’s alphas and intraclass correlation coefficients ≥ 0.70). Other important measures will be collected using validated measures in their official French versions: side effects (Functional Assessment of Cancer Therapy—Endocrine Symptoms (FACT-ES) [51]), fear of cancer recurrence (Fear of Cancer Recurrence Inventory [52]), beliefs about medication (Beliefs about Medicine Questionnaire – adjuvant endocrine therapy [53]), patient-pharmacist relationship (Consultation and Relational Empathy (CARE) measure [54, 55]), and quality of life (Functional Assessment of Cancer Therapy – Breast (FACT-B) [56]).

We will appraise the program's preliminary effects on *AET adherence* (AET initiation, implementation, and persistence) [57] through self-reported measures (i.e., using questions developed by our team and the Medication Adherence Report Scale (MARS) [58]) and calculate it using pharmacy claims for the 6-month follow-up. Data on drug identifier, delivery date, prescribed regimen, and duration of supply will be used to calculate AET initiation, non-persistence, and implementation for any AET, not just the one initially prescribed. Non-persistence will be defined as not claiming an AET at the expected date, allowing for a 90-day permissible gap. Implementation will be defined through the proportion of days covered (PDC) by any AET divided by the number of days of follow-up [59].

Information on the *feasibility of a large-scale cluster RCT* will be gathered through recruitment rates (% of eligible and enrolled pharmacists and patients, time for recruitment), recruitment channels, reasons for refusal, randomization process, cluster size, and frequency of contact with the research team. *Characteristics* of pharmacies (e.g., number of daily prescriptions delivered) and pharmacists (e.g., years of experience), as well as sociodemographic and medical characteristics of the patients (e.g., age, other breast cancer treatment received) will be collected in the baseline questionnaire. *Co-interventions* (i.e., other services received regarding AET) will be collected in the 6-month questionnaire completed by patients. *Costs* of program development and delivery will be documented by the research team.

### Data collection and management

Quantitative outcomes will be collected through the Research Electronic Data Capture (REDCap) online platform [60]. Pharmacists and participating patients will be asked to complete two online questionnaires: one before randomization and another at the end of the 6-month follow-up. Non-responders will be reminded using email and telephone. Qualitative data will be collected at the end of the study through semi-structured interviews. These interviews will be conducted by telephone or Teams and audio-recorded. Participants’ confidential information will be managed according to best practices, stored on a secured server, and kept in password-protected documents accessible only to a limited number of research team members who have signed a confidentiality agreement.

### Progression criteria

The following indicators will be used to determine the pilot study’s acceptability and feasibility: ≥ 50% of eligible patients agreeing to participate; ≥ 70% of recruited pharmacists having completed the training and using the program tools; ≥ 75% of pharmacists and patients deeming the program acceptable and potentially beneficial. For the objective related to the program’s preliminary effects, we will consider that an effect size of ≥ 0.50 on factors hypothesized to influence adherence, such as intention, as indicating that the program has the potential to enhance AET adherence, since a moderate to large effect on such factors is likely to produce a change in the targeted behavior (i.e., AET adherence [61]).

### Sample size

We estimated that most clusters would include between 1 and 3 patients (mean = 2) and that we would be able to recruit approximately 33 pharmacies and 66 patients per group. To assess the precision of our acceptability and feasibility indicators, we calculated Wald 95% confidence intervals (CI) [62] taking into account an intra-cluster correlation (ICC) of 0.02 for patients from the same pharmacy [63]. For example, for the acceptability indicator defined by a participation rate of at least 50% of eligible patients, a sample size of 132 patients will lead to a 95% CI of 41.5% to 58.5%. For one of our main feasibility indicators, which is at least 70% of pharmacists completing the training, a sample size of 33 pharmacists will result in a 95% CI of 54.4% to 85.6%. Finally, for the indicator assessing whether at least 75% of pharmacists and patients deem the program acceptable and potentially beneficial, a sample size of 33 pharmacists and 66 patients will result in a 95% CI of 60.2% to 89.8% and 64.4% to 85.6%, respectively. This pilot study is not designed to assess the program’s efficacy on adherence [64]. However, we will be able to explore the program’s preliminary effects on factors expected to influence AET adherence. The anticipated sample size will enable us to detect moderate to large effect sizes (*d* ≥ 0.50) (mean of 2 patients per cluster; ICC = 0.02 [63]; correlation of 0.5 between baseline and 6-month follow-up [65]; power = 0.80; α = 0.05). For the qualitative phase, we will determine the final number of semi-structured interviews to be conducted for each group (pharmacists and patients) using data saturation (i.e., when no new information is provided by the interviews). From our previous work conducted among these groups [66–68], saturation may be reached after 15 to 20 interviews.

### Analyses

Descriptive statistics for pharmacies, pharmacists, and patients in the PACHA program and UC group will be reported and compared. Acceptability and feasibility indicators such as recruitment and retention rates will be evaluated using percentages or means and 95% CI [69]. To explore preliminary effects on factors expected to influence AET adherence, generalized estimating equation (GEE) regression models will be used to compare patterns of change in scores of factors influencing AET adherence between the two groups over time. The group-by-time interaction *p*-value will be used to test the differences between the two groups. Effect sizes will be estimated using Cohen’s *d*. The required sample size for a large-scale cluster RCT will be estimated using the PDC, its variability, and ICC. Missing data will be handled using various strategies according to the variables. For instance, mean score imputation (if at least half of the items are completed) will be applied to scales, while listwise deletion will be used for other variables [70]. For qualitative data, thematic analysis of the interviews will be conducted [71, 72]. Since this is a pilot study, analyses will be performed at the end of the study, and no subgroup analyses will be performed.

### Data monitoring

A committee composed of breast cancer and pharmacy practice experts will be involved in all key steps of the study. The committee contributed to refining this pilot study and will continue to monitor its progress, suggest new approaches when needed, and facilitate knowledge transfer and application. 

### Harms and safety

Pharmacists in the PACHA program group will complete the web training and apply the program tools, but their practice will remain consistent with current provincial pharmacy practice standards. Pharmacists in the UC group will provide usual care. Thus, there is no risk to the safety of participating patients and pharmacists.

### Auditing

Audits may be conducted randomly by the quality assurance team at the CHU de Québec–Université Laval Research Centre.

### Knowledge dissemination

The results, methods, and lessons from this pilot study will be shared with the scientific community through presentations at scientific conferences and publications in open-access scientific journals. We will also present the results and prepare written summaries for community pharmacists and their associations.

## Discussion

The goal of this pilot study is to assess the acceptability, feasibility of implementation, and preliminary effects of the PACHA program, a community pharmacy-based program designed to support patients prescribed AET for hormone-sensitive breast cancer. Following a mixed-methods design, multiple quantitative measures and indicators will be collected, along with qualitative semi-structured interviews conducted with participating patients and pharmacists.

In planning this pilot study, we anticipated several challenges and addressed them where possible. First, to prevent contamination of the intervention, we chose a cluster-RCT design. Such design implies that program training and tools are available exclusively to pharmacies randomized to the PACHA program group. However, since the training holds strong appeal for pharmacists, we decided to grant access to the training to those in the control group after the 6-month follow-up period. This decision will potentially have a positive effect on the control group retention rate. Second, while pharmacists and patients cannot be blinded to the intervention, the analyses will be performed in a blinded fashion. Third, evaluating such a complex, multifaceted intervention requires taking into account variables at the pharmacy, pharmacist, and patient levels. For this reason, we conceived our outcome assessment plan based on the Framework for the Implementation of Services in Pharmacy [26] and the Theory of Planned Behavior [50]. We also drew on our extensive program development and pre-testing phase [24, 32]. Finally, following the COVID-19 pandemic and based on feedback from community pharmacists, we decided to modify the delivery mode of our intervention, moving from in-person consultations to telephone consultations, and from mainly printed material to mostly web-based material [24]. Compared to in-person consultations, telephone consultations were deemed more flexible and feasible by our pharmacist informants. This approach is also more efficient in a context where patients do not always go to their community pharmacy to get their medication (e.g., most pharmacies offer medication delivery). We also consider that both the web-based training and resources are major assets of the PACHA program in the current post-COVID 19 context and could facilitate future dissemination and implementation of the program.

The development and testing of innovative approaches for enhancing AET adherence are needed to attain the full benefits of this treatment. This project will advance knowledge on anticancer treatment optimization by pilot testing, which, to our knowledge, is the first community-pharmacy-based program specifically designed to enhance adherence to AET. Results from this pilot study will indicate if involving community pharmacists to support cancer survivors with oral anticancer medications is acceptable, feasible, and promising from the perspectives of breast cancer survivors and community pharmacists. The results will also include several outcomes related to patients’ experiences with the treatment, capturing this important patient-centered perspective. These quantitative and qualitative results will be used to guide our decisions on refining the program content and implementation plan, as well as conducting and designing a large-scale cluster-RCT to assess its effectiveness and cost-effectiveness. If both effective and cost-effective, this program could contribute to efforts made to support breast cancer survivors and improve their quality of life. It could also be adapted for other oral anticancer medications.

## Data Availability

Not applicable—paper is a protocol.

## Abbreviations

AET: Adjuvant endocrine therapy
CARE: Consultation and Relational Empathy measure
FACT-B: Functional Assessment of Cancer Therapy–Breast
FACT-ES: Functional assessment of cancer therapy–Endocrine Symptoms
ICC: Intra-cluster correlation
GEE: Generalized estimation equation
MARS: Medication Adherence Report Scale
MI: Motivational Interviewing
PACHA: *Programme en Pharmacie pour l’ACcompagnement des femmes ayant de l’Hormonothérapie Adjuvante* (Pharmacy program for supporting women undergoing adjuvant endocrine therapy)
OPQ: *Ordre des pharmaciens du Québec*
PDC: Proportion of days covered
RCT: Randomized controlled trial
REDCap: Research electronic data capture
UC: Usual care
